# Child work and labour among orphaned and abandoned children in five low and middle income countries

**DOI:** 10.1186/1472-698X-11-1

**Published:** 2011-01-13

**Authors:** Rachel Whetten, Lynne Messer, Jan Ostermann, Kathryn Whetten, Brian Wells Pence, Megan Buckner, Nathan Thielman, Karen O'Donnell

**Affiliations:** 1 Center for Health Policy, Duke Global Health Institute, Duke University, Durham, North Carolina, USA; 2 Duke Global Health Institute, Duke University, Durham, North Carolina, USA; 3 Department of Medicine, Division of Infectious Diseases and International Health, Duke University, Durham, North Carolina, USA; 4 Department of Community and Family Medicine, Duke University, Durham, North Carolina, USA; 5 Departments of Psychiatry and Pediatrics, Duke University, Durham, NC, USA; 6 Center for Child and Family Health, Duke University, Durham, NC, USA; 7 Membership of The Positive Outcomes for Orphans (POFO) Research Team is provided in the Acknowledgements

## Abstract

**Background:**

The care and protection of the estimated 143,000,000 orphaned and abandoned children (OAC) worldwide is of great importance to global policy makers and child service providers in low and middle income countries (LMICs), yet little is known about rates of child labour among OAC, what child and caregiver characteristics predict child engagement in work and labour, or when such work infers with schooling. This study examines rates and correlates of child labour among OAC and associations of child labour with schooling in a cohort of OAC in 5 LMICs.

**Methods:**

The Positive Outcomes for Orphans (POFO) study employed a two-stage random sampling survey methodology to identify 1480 single and double orphans and children abandoned by both parents ages 6-12 living in family settings in five LMICs: Cambodia, Ethiopia, India, Kenya, and Tanzania. Regression models examined child and caregiver associations with: any work versus no work; and with working <21, 21-27, and 28+ hours during the past week, and child labour (UNICEF definition).

**Results:**

The majority of OAC (60.7%) engaged in work during the past week, and of those who worked, 17.8% (10.5% of the total sample) worked 28 or more hours. More than one-fifth (21.9%; 13% of the total sample) met UNICEF's child labour definition. Female OAC and those in good health had increased odds of working. OAC living in rural areas, lower household wealth and caregivers not earning an income were associated with increased child labour. Child labour, but not working fewer than 28 hours per week, was associated with decreased school attendance.

**Conclusions:**

One in seven OAC in this study were reported to be engaged in child labour. Policy makers and social service providers need to pay close attention to the demands being placed on female OAC, particularly in rural areas and poor households with limited income sources. Programs to promote OAC school attendance may need to focus on the needs of families as well as the OAC.

## Background

Child labour is an issue of international concern as it can have negative effects on the child's health, educational achievement, and quality of life. The International Labour Organization (ILO) estimates that 218 million children are engaged in child labour [1]. While some work by children may be beneficial [2], there is consensus that physically harmful work and/or working long hours (e.g. "child labour") lead to decreased educational achievement and potentially decreased physical and mental wellbeing [3] and negatively influence children's ability to reach their potential as adults [4]. In recent years, positive responses to national and international policy efforts to limit child labour have raised hopes that it may be possible to reduce child labour and to mitigate the negative effects of such labour on children [5-7]. However, there is still debate among international organizations concerning the definition of "child labour" [8] and little empirical data from multi-country studies exist to identify points at which work interferes with education and health.

An estimated 143,000,000 children are single or double orphans, making their wellbeing and future productivity a concern for international and national policy makers and local providers. Poverty is known to predict child labour [9]; and parental illness or death, whether from HIV or other causes, amplifies these effects. Households may incur significant medical costs and lose a source of income, and children may be called upon to care for dying relatives, to take on additional household chores, or to supplement the household's income by engaging in income-generating activities. Little is known about the work and labour patterns of orphaned and abandoned children (OAC) in LMIC and how their work interferes with school attendance and wellbeing.

One limitation of the research literature concerning child labour is the lack of a uniform definition [2,6,10-15]. Definitions of "child labour" range from including all work, including household chores, childcare and other domestic work, and wage work in the formal sector; to including only economic market activity. The number of hours per week of work assumed to put a child at risk also varies from study to study. The lack of a universally-accepted, working definition of child labour makes it difficult to compare studies across cultures and across time. UNICEF suggests a definition of child labour being 28 hours or more per week of any kind of work, and when work is for pay, the number of hours allowed is less [16]. Both UNICEF and the ILO believe it is important to include both paid and unpaid work because by including only paid work, one prioritizes work outside the home for money and ignores the burden that reportedly falls largely on girl children within the household to support the family by working long hours for which they are not paid. Both working outside the home for pay and working long hours at home can result in fewer hours for attending school and studying.

Examining data from a sample of OAC aged 6 to 12 who were randomly selected from 6 sites in 5 LMICs, this study describes rates of work among the OAC, correlates of work with child and caregiver characteristics, and cut-offs at which child work interferes with schooling. The child work categories that are compared are: working any hours; working 1 to 20 hours, 21 to 27 hours; and 28 or more hours; and the UNICEF definition of child labour of 28 hours or more of any work, youth younger than age 12 working for financial compensation, or youth aged 12 or over working 14 or more hours per week for pay.

## Methods

### Study description

The Positive Outcomes for Orphans (POFO) study is a longitudinal cross-cultural research study designed to identify characteristics of care, across diverse care structures and cultures, associated with better child outcomes. Two-stage random sampling survey methodology identified a sample of 1,480 OAC ages 6-12 living in family setting in 309 randomly selected geographical community clusters in six study sites across five LMICs: Cambodia, India (two sites), Kenya, Tanzania, and Ethiopia. Additional study details have been published elsewhere [17,18].

### Study sample

#### Country Selection

From a group of 13 countries in which members of the research team had existing relationships with grassroots organizations with an interest in the proposed research, five countries were selected that were culturally, historically, ethnically, religiously, politically, and geographically diverse from each other. In India, two sites, Hyderabad and Nagaland, were chosen due to their vastly different populations represented in terms of religion, geography, income levels and political histories. Political boundaries were used to define the study area at each site [17,18].

#### Community Sampling Area Selection

The primary community sampling aim was to select an unbiased sample of family-based care settings. In each of the six study areas, 50 sampling areas ("clusters") were randomly selected; in 4 of the 6 sites clusters were equally split between rural and urban areas, while in 2 the entire site was urban. *At each of the six study sites, clusters were defined as villages, streets, or similar geographic units that were randomly selected from the respective next larger politically or administratively defined area (e.g., wards, kebeles, or other areas comparable to counties or zip codes in the United States). These areas in turn were randomly selected from all such areas within the boundaries of each study site. The number of clusters per study site ranged from 47 to 58 for a total of 311 clusters*.

#### Selection of Family-dwelling OAC

A family-dwelling OAC was defined as a single or double orphan or a child abandoned by both parents who was living in a family situation as opposed to living in an institution or on the streets. In each community sampling area, up to five eligible children were selected, either randomly from available lists or through a house-to-house census conducted until five households with age-eligible children were identified. In households with multiple age-eligible children, the child whose first name started with the earliest letter in the alphabet was selected for study inclusion. In total, 1,480 OAC living in families were enrolled in the study.

#### Caregiver Selection

The children's (self-identified) primary caregivers were asked to respond to surveys about themselves and the children. In total, 1,480 community-based caregivers participated in the assessments.

#### Interviewer Training

One local male and female interviewer and a lead investigator from each site were trained on study protocol and procedures. A week-long training took place at a central location with all interviewers and primary investigators present. Interviewers were certified after repeated direct observation or video taping of interviews with local non-study children. Cross-site training and site visits with interviewer observation ensured accuracy and consistency across interviewers and study sites.

#### Data collection protocol

Baseline data collection started between May 2006 and April 2007, depending on the study site, and continued for approximately six months. We have no reason to believe that the variation in the enrolment timeframe across study sites biased the results. Informed consent was obtained from each participating caregiver. Assent was given by all participating children. Interviews were conducted with children and caregivers using their native language in the child's residence. Baseline instruments collected information about: 1) children aged 6 to 12 residing in family settings that had at least one parent who had died or had been abandoned by both parents; and 2) the children's primary caregivers. Age inclusion criteria were based on survey instrument validity and pilot testing. Ethical approval was provided by the Duke University Institutional Review Board (IRB) and by local and national IRBs in all participating countries.

### Study measures

#### Child measures

Baseline child-level characteristics were collected from caregivers during the interview. Caregivers reported on child demographics (gender, age); school attendance; relationship to caregiver (including biological child, other relative, or non-relative) and the child's general health (using one item from the Medical Outcomes Study Short Form [19]), with response options of "very good," "good," "fair," "poor," "very poor". Caregivers also reported if children worked ("During the past week, did the child do any kind of work for someone who is not a member of this household;" responses: "yes, paid;" "yes, unpaid," "no"), engaged in household chores ("During the past week, did the child do any household chores, such as farming, childcare, or other housework;" responses: "yes," "no," "don't know"), and approximately how many hours per week the child spent in each activity.

#### Caregiver measures

During the same baseline interviews, caregivers reported on their own demographics (gender, age, marital status); education (highest grade completed); general health (as above); whether they earn an income ("Do you earn an income;" "yes" or "no"); and the number of children they care for. A wealth index comparable to that used in the Demographic and Health Surveys was constructed to summarize assets and physical characteristics of each participating household [20].

#### Work and Labour variable construction

The outcome variables for this analysis were child work and child labour. Children were coded as having engaged in any work if their caregiver reported that they engaged in either work outside the home or in household chores. Hours spent working were categorized as either <21 hours, 21 to 27 hours; or child labour, defined according the UNICEF definitions of 28 hours or more of any work, youth younger than age 12 working for financial compensation, or youth aged 12 or older working 14 or more hours per week for pay. The POFO study did not assess hazardous work, which is included in UNICEF's definition of child labour, making labour estimates from this study conservative. The cut-off of 21 hours was included in the analyses because ILO suggested this as an alternative limit for the definition of child labour.

#### Analyses

Logistic regression was used to predict the dichotomous outcome of any work vs. no work; multinomial logistic regression models were used to identify associations with the four work and labour categories: no work, <21 hours per week, 21-27 hours per week, and child labour, as defined above. Covariates alternatively included child characteristics, caregiver characteristics, or child and caregiver characteristics. To account for the stratified sampling and clustering of children within sites, logistic models were estimated with randomly-distributed site-specific intercepts; multinomial logistic regression models included site fixed effects and were estimated with robust standard errors. Analyses were conducted using Stata version 11.1.

## Results

Most of the children (60.7%) were reported to have engaged in work during the past week; of those who worked, 11.5% worked from 21 to 27 hours per week, 17.8% worked for 28 or more hours per week, and 21.9% met the UNICEF definition for child labour (Table 1). Half of the OAC (52.4%) were male and the majority (94%) attended school. Over half were single orphans living with the remaining biological parent (55.2%), while other relatives were caring for most of the remaining OAC (43.1%). Most children were reported to be in good or very good health (63.8%), with 8.1% in poor or very poor health.

**Table 1:** Child and caregiver characteristics of the POFO sample

	CHILD CHARACTERISTICS	(n = 1,473)	
**(categorical)**			
	**Number**	**Percent**	**Missing**
**Gender**			0
Male	772	52.4	
Female	701	47.6	
**Age**			0
6-7 years	384	26.1	
8-10 years	754	51.2	
11-12 years	335	22.7	
**Relation to caregiver**			0
Biological child	813	55.2	
Other relative	635	43.1	
Non-relative	25	1.7	
**Health**			19
Very good, good	928	63.8	
Good	408	28.1	
Poor, very poor	118	8.1	
**Work/Labour status**			7
Any Work	890	60.7	
No Work	576	39.3	
**Categorized hours worked**			0
<21 hours per week	618	70.8	
21-27 hours per week	100	11.5	
28 + hours per week	155	17.8	
UNICEF child labour*	191	21.9	
**Education**			
Yes	1361	94.0	
No	86	5.9	
			
**CAREGIVER CHARACTERISTICS**		(n = 1464)	
(categorical)			
	**Number**	**Percent**	**Missing**
**Gender**			4
Male	215	14.7	
Female	1245	85.3	
**Age**			61
<18 years	10	0.7	
18-35 years	510	36.4	
36-55 years	629	44.8	
56 + years	254	18.1	
**Marital status**			5
Married	385	26.4	
Widowed	894	61.3	
Never married & Other	180	12.3	
**Health**			20
Very good, good	678	47.0	
Fair	479	33.2	
Poor, very poor	287	19.9	
**Earn an income**			10
Yes	1033	71.1	
No	421	29.0	
(continuous)	**Mean (sd)**	**Range**	
**Age (in years)**	42.5 (13.6)	11 to 89	
**Education (in years)**	5.3 (4.3)	0 to 18	
**Children cared for (number)**	2.8 (2.1)	0 to 19	

* The UNICEF child labour variable includes = (work 28 or more hours per week) + (any youth <12 years of age working any number of hours for pay) + (any youth 12+ years of age or older working 14 or more hours per week for pay). The POFO dataset does not identify hazardous work, therefore potentially misses some children engaged in child labour.

The majority of caregivers were female (85.3%). On average caregivers were42.5 years old, and more than half were widowed (61.3%). Many caregivers reported being in good or very good health (46.5%), while one-fifth (19.9%) reported being in poor or very poor health. More than one-quarter reported no income (29.0%). The mean number of years of education reported by these caregivers was 5.3 and they were caring for three children, on average (sd = 2.1).

Analyses examining child characteristics that were associated with work and labour found that being female and of older age were significantly associated with all work and labour comparisons with significance levels of 0.05 or lower (Table 2). Rates of child labour ranged from 5.1% among 6 and 7 year old boys to 20.4% among girls aged 11 or 12. While poor or very poor health of the child, as reported by the caregiver, was associated with any work versus no work (adjusted odds ratio (AOR) = 0.42; 95% Confidence Interval (95% CI): 0.24, 0.76) and working <21 hours versus no work (AOR = 0.38; 95% CI: 0.20, 0.70), child health was not associated with child labour. The relationship of the child to the caregiver was not associated with the level of work or child labour

**Table 2:** Multivariable associations between child characteristics, work status and labor

		**Any work **^**1**^	**<21 hrs **^**2**^	**22-27 hrs **^**2**^	**Labor **^**2**^
Gender	Male	1.00	1.00	1.00	1.00
		*(reference)*	*(reference)*	*(reference)*	*(reference)*
	Female	1.95**	1.93**	1.94*	2.16**
		[1.46 - 2.61]	[1.42 - 2.61]	[1.17 - 3.21]	[1.43 - 3.27]
Relationship	Biological child	1.00	1.00	1.00	1.00
		*(reference)*	*(reference)*	*(reference)*	*(reference)*
	Other	1.11	1.08	1.38	1.07
		[0.83 - 1.48]	[0.79 - 1.47]	[0.83 - 2.32]	[0.71 - 1.61]
School enrollment	Enrolled	1.00	1.00	1.00	1.00
		*(reference)*	*(reference)*	*(reference)*	*(reference)*
	Not enrolled	2.14*	1.72	1.66	5.04**
		[1.18 - 3.90]	[0.93 - 3.16]	[0.51 - 5.41]	[2.35 - 10.84]
Caregiver reported	Very good or good	1.00	1.00	1.00	1.00
health of the child		*(reference)*	*(reference)*	*(reference)*	*(reference)*
	Fair	0.85	0.9	0.93	0.64
		[0.57 - 1.27]	[0.59 - 1.36]	[0.46 - 1.89]	[0.37 - 1.10]
	Poor or very poor	0.42**	0.38**	0.53	0.53
		[0.24 - 0.76]	[0.20 - 0.70]	[0.19 - 1.49]	[0.25 - 1.12]
Age	6-7	1.00	1.00	1.00	1.00
		*(reference)*	*(reference)*	*(reference)*	*(reference)*
	8-10	3.45**	3.08**	4.45**	5.50**
		[2.44 - 4.87]	[2.15 - 4.40]	[2.18 - 9.11]	[3.10 - 9.77]
	11-12	5.85**	4.85**	10.64**	9.82**
		[3.80 - 9.02]	[3.07 - 7.64]	[4.86 - 23.27]	[5.14 - 18.76]
Location	Urban	1.00	1.00	1.00	1.00
		*(reference)*	*(reference)*	*(reference)*	*(reference)*
	Rural	2.38**	2.12**	2.96**	2.94**
		[1.65 - 3.45]	[1.46 - 3.09]	[1.70 - 5.15]	[1.84 - 4.69]

*, **, and *** denote significance at the 0.05, 0.01, and 0.001 levels, respectively

^1 ^relative to no work; estimates from a logistic regression model controlling for site fixed effects

^2 ^relative to no work; estimates from a multinomial logistic regression model controlling for site fixed effects

Not attending school was significantly associated with child labour: OAC who were not attending school were 4 times more likely to engage in child labour than those in school (AOR = 5.04; 95% CI: 2.35, 10.84), and children who were engaged in child labour were twice as likely to not attend school compared to children who worked fewer than 28 hours (10.4% vs. 5.3%, p < 0.001, not shown). While school attendance was significantly associated with working versus not working (AOR = 2.14; 95% CI: 1.18, 3.90), it was not associated with working <21 hours or working 21 to 27 hours, indicating that the significant result is driven by children engaged in child labour.

Table 3 presents associations between caregiver characteristics and child work and labour categories. Living in a rural area was associated with all categories of child work and labour at significance levels of 0.01 or below. The odds ratios increase with each category; OAC living in rural areas were more than 2.5 times more likely to engage in child labour than those in urban areas (AOR = 3.60; 95% CI: 2.02, 6.41). Lower wealth was associated with all categories except working 21-27 hours. Caregivers not earning an income more than doubled the likelihood of child labour (AOR = 2.06; 95% CI: 1.14, 3.75).

**Table 3:** Multivariable associations between caregiver characteristics, child work status and child labor

		**Any work **^**1**^	**<21 hrs **^**2**^	**22-27 hrs **^**2**^	**Labor **^**2**^
Caregiver gender	Male	1.00	1.00	1.00	1.00
		*(reference)*	*(reference)*	*(reference)*	*(reference)*
	Female	1.02	0.91	1.27	1.48
		[0.64 - 1.62]	[0.55 - 1.48]	[0.53 - 3.04]	[0.68 - 3.21]
Caregiver age	18-35	1.00	1.00	1.00	1.00
		*(reference)*	*(reference)*	*(reference)*	*(reference)*
	36-55	0.74	0.72	0.65	0.82
		[0.51 - 1.07]	[0.49 - 1.06]	[0.34 - 1.25]	[0.46 - 1.46]
	56+	0.69	0.65	0.62	0.8
		[0.39 - 1.22]	[0.36 - 1.20]	[0.25 - 1.52]	[0.35 - 1.82]
Marital status	Married	1.00	1.00	1.00	1.00
		*(reference)*	*(reference)*	*(reference)*	*(reference)*
	Widowed	0.74	0.85	0.65	0.46**
		[0.50 - 1.11]	[0.56 - 1.30]	[0.32 - 1.32]	[0.26 - 0.81]
	Other	0.76	0.87	0.62	0.54
		[0.43 - 1.35]	[0.48 - 1.59]	[0.19 - 2.01]	[0.21 - 1.38]
Caregiver health	Very good or good	1.00	1.00	1.00	1.00
		*(reference)*	*(reference)*	*(reference)*	*(reference)*
	Fair	1.44	1.47	1.85	1.16
		[0.96 - 2.17]	[0.96 - 2.25]	[0.89 - 3.84]	[0.65 - 2.08]
	Poor or very poor	0.67	0.66	0.68	0.71
		[0.41 - 1.11]	[0.40 - 1.08]	[0.28 - 1.67]	[0.34 - 1.45]
Caregiver education	Less than 7 years	1.00	1.00	1.00	1.00
		*(reference)*	*(reference)*	*(reference)*	*(reference)*
	7 years or more	1.24	1.19	1.21	1.49
		[0.85 - 1.81]	[0.81 - 1.74]	[0.63 - 2.33]	[0.83 - 2.70]
Earning an income	Yes	1.00	1.00	1.00	1.00
		*(reference)*	*(reference)*	*(reference)*	*(reference)*
	No	1.05	0.87	1.41	2.06*
		[0.70 - 1.60]	[0.56 - 1.37]	[0.64 - 3.09]	[1.14 - 3.75]
Wealth index		0.54**	0.58**	0.61	0.34**
		[0.39 - 0.74]	[0.43 - 0.80]	[0.37 - 1.02]	[0.22 - 0.55]
# of children caring for		1.04	1.04	1.06	0.95
		[0.94 - 1.14]	[0.95 - 1.15]	[0.91 - 1.24]	[0.82 - 1.09]
Location	Urban	1.00	1.00	1.00	1.00
		*(reference)*	*(reference)*	*(reference)*	*(reference)*
	Rural	2.79**	2.44**	3.27**	3.60**
		[1.83 - 4.25]	[1.60 - 3.73]	[1.71 - 6.26]	[2.02 - 6.41]

*, **, and *** denote significance at the 0.05, 0.01, and 0.001 levels, respectively

^1 ^relative to no work; estimates from a logistic regression model controlling for site fixed effects

^2 ^relative to no work; estimates from a multinomial logistic regression model controlling for site fixed effects

When child and caregiver characteristics were controlled for jointly, OAC who had missed school were 3 times more likely to be engaged in child labour (Table 4; AOR = 4.04; 95% CI: 1.37, 11.94). Missing school was not associated with other levels of work. Being female was associated with twice the likelihood of work and child labour across all comparisons with p-values of 0.01. Similarly, older child age was positively associated with work and labour across all comparisons with p-values of less than 0.001. Older caregiver age was associated with a lower likelihood of working versus not working and working <21 hours versus not working (p < 0.05). Rural living was associated with less work in all comparisons (p < 0.001), wealth was associated with work and labour in all comparisons except 21 to 27 hours versus no work (p < 0.01); not having an income was associated with child labour only (AOR = 2.26; 95% CI: 1.20, 4.23; p < 0.05).

**Table 4:** Multivariable associations between child and caregiver characteristics, work status and labor

		**Any work **^**1**^	**<21 hrs **^**2**^	**22-27 hrs **^**2**^	**Labor **^**2**^
Child gender	Male	1.00	1.00	1.00	1.00
		*(reference)*	*(reference)*	*(reference)*	*(reference)*
	Female	2.23**	2.27**	2.46**	2.06**
		[1.57 - 3.18]	[1.57 - 3.28]	[1.37 - 4.43]	[1.23 - 3.46]
Relationship	Biological child	1.00	1.00	1.00	1.00
		*(reference)*	*(reference)*	*(reference)*	*(reference)*
	Other	1.54	1.51	2.34	1.23
		[0.92 - 2.57]	[0.91 - 2.52]	[0.91 - 6.04]	[0.60 - 2.53]
School enrollment	Enrolled	1.00	1.00	1.00	1.00
		*(reference)*	*(reference)*	*(reference)*	*(reference)*
	Not enrolled	2.02	1.71	1.94	4.04*
		[0.94 - 4.35]	[0.82 - 3.56]	[0.48 - 7.86]	[1.37 - 11.94]
Caregiver reported	Very good or good	1.00	1.00	1.00	1.00
health of the child		*(reference)*	*(reference)*	*(reference)*	*(reference)*
	Fair	1.27	1.28	1.88	1.05
		[0.75 - 2.16]	[0.74 - 2.21]	[0.80 - 4.42]	[0.52 - 2.11]
	Poor or very poor	0.53	0.49	0.64	0.55
		[0.26 - 1.07]	[0.24 - 1.03]	[0.18 - 2.20]	[0.22 - 1.37]
Child age	6-7	1.00	1.00	1.00	1.00
		*(reference)*	*(reference)*	*(reference)*	*(reference)*
	8-10	4.05**	3.55**	6.84**	6.70**
		[2.68 - 6.13]	[2.33 - 5.41]	[2.79 - 16.78]	[3.24 - 13.84]
	11-12	4.80**	4.09**	10.87**	7.17**
		[2.90 - 7.92]	[2.39 - 6.98]	[4.11 - 28.73]	[3.26 - 15.77]
Caregiver gender	Male	1.00	1.00	1.00	1.00
		*(reference)*	*(reference)*	*(reference)*	*(reference)*
	Female	1.2	1.34	1.06	0.79
		[0.73 - 1.97]	[0.79 - 2.28]	[0.44 - 2.56]	[0.35 - 1.77]
Caregiver age	18-35	1.00	1.00	1.00	1.00
		*(reference)*	*(reference)*	*(reference)*	*(reference)*
	36-55	0.68	0.67	0.57	0.77
		[0.46 - 1.02]	[0.44 - 1.02]	[0.28 - 1.13]	[0.42 - 1.42]
	56+	0.48*	0.45*	0.35	0.66
		[0.24 - 0.94]	[0.22 - 0.94]	[0.12 - 1.02]	[0.26 - 1.68]
Marital status	Married	1.00	1.00	1.00	1.00
		*(reference)*	*(reference)*	*(reference)*	*(reference)*
	Widowed	1	1.13	1.22	0.51
		[0.58 - 1.73]	[0.66 - 1.94]	[0.49 - 3.04]	[0.25 - 1.05]
	Other	1.01	1.14	0.91	0.66
		[0.54 - 1.87]	[0.59 - 2.21]	[0.26 - 3.26]	[0.24 - 1.81]
Caregiver health	Very good or good	1.00	1.00	1.00	1.00
		*(reference)*	*(reference)*	*(reference)*	*(reference)*
	Fair	1.51	1.56	1.79	1.23
		[0.97 - 2.36]	[0.97 - 2.52]	[0.81 - 3.96]	[0.65 - 2.32]
	Poor or very poor	0.85	0.83	0.87	0.97
		[0.50 - 1.47]	[0.48 - 1.46]	[0.34 - 2.23]	[0.44 - 2.10]
Caregiver education	Less than 7 years	1.00	1.00	1.00	1.00
		*(reference)*	*(reference)*	*(reference)*	*(reference)*
	7 years or more	1.03	1.02	1.02	1.06
		[0.97 - 1.09]	[0.96 - 1.08]	[0.92 - 1.12]	[0.97 - 1.16]
Earning an income	Yes	1.00	1.00	1.00	1.00
		*(reference)*	*(reference)*	*(reference)*	*(reference)*
	No	1.12	0.95	1.52	2.26*
		[0.72 - 1.76]	[0.59 - 1.52]	[0.67 - 3.44]	[1.20 - 4.23]
Wealth index		0.55**	0.59**	0.63	0.36**
		[0.39 - 0.78]	[0.42 - 0.83]	[0.36 - 1.09]	[0.22 - 0.59]
# of children caring for		1.04	1.05	1.06	0.96
		[0.94 - 1.16]	[0.94 - 1.17]	[0.91 - 1.25]	[0.83 - 1.12]
Location	Urban	1.00	1.00	1.00	1.00
		*(reference)*	*(reference)*	*(reference)*	*(reference)*
	Rural	3.12**	2.76**	3.91**	3.88**
		[1.98 - 4.90]	[1.74 - 4.36]	[1.94 - 7.89]	[2.12 - 7.10]

*, **, and *** denote significance at the 0.05, 0.01, and 0.001 levels, respectively

^1 ^relative to no work; estimates from a logistic regression model controlling for site fixed effects

^2 ^relative to no work; estimates from a multinomial logistic regression model controlling for site fixed effects

## Discussion

The majority of OAC aged 6-12 in this study engaged in some sort of work during the previous week (60.7%) and of those who worked, 17.8% (10.5% of the total sample) did so for 28 or more hours. More than one-fifth (21.9%, 13% of the total sample) met UNICEF's child labour definition, excluding hazardous work. The omission of hazardous work from the definition of child labour, the comparatively young age, and high rates of urban residence among OAC in the POFO cohort, suggest that rates of child labour among OAC in the six study sites may be higher than general estimates that indicate that 16% of children aged 5 to 14 in LMICs engage in child labour [21].

Child work was associated with significantly lower rates of school attendance when the work exceeded 27 hours per week. This relationship was even stronger among OAC younger than 12 years working for pay and OAC ages 12 or above who worked 14 or more hours per week for pay. While these findings support UNICEF child labour definition as a standard for policy makers looking to eliminate child work that might interfere with schooling, it is possible that the lower limit of 21-27 hours suggested by some was not significant because the study cohort is comprised of only OAC who predominantly reside in poorer or otherwise disadvantaged households. The estimated associations between labour and schooling may thus represent estimates at the margin.

The importance of rural vs. urban residence, and income and wealth of the household, indicate the need for policies and programs designed to reduce child labour to focus on family and household support. While wealth was associated with all categories of work and labour, lack of income was associated only with child labour in the full model. It is important to recognize that a household may be very poor, or have little wealth, but the loss of an income earning adult may be what makes a family decide that an OAC must engage in child labour and stop attending school.

The finding that female children were more than twice as likely to be engaged in child labour provides further evidence that that girl children face a particularly heavy burden with respect to household duties, childcare and other kinds of labouring. The finding supports the argument made by child protection and policy making organizations that when unpaid domestic "chores" are not counted as labour, we risk missing the large burden being placed on children, particularly girl children, which can interfere with their educational attainment and future wellbeing.

One unique aspect of this paper is its explicit focus on labour among orphans and abandoned children (OAC); to our knowledge, it is the first of its kind to do so. While point estimates suggest slightly higher odds of child work or labour if the child was not living with a biological parent, the lack of statistical significance suggests being with a biological relative was not protective against labour or work.

Much of the child labour literature focuses on educational attainment and school attendance, as it is widely believed that child labour interferes with schooling [5,22,23]. Research conducted in five culturally distinct countries examined the relationship of school attendance, grade attainment and reported working hours, defined as both market and non-market related activity. The authors noted an inverse association with both *market-activity *defined as..."activities leading to the production of goods and services which are intended for sale or sold on the market" [15], *and non-market activity *defined as... "production of goods and services for members of a household for household use", e.g. fetching water, meal preparation, child care, etc... [15] working hours and school attendance rates in all countries, with drops in attendance around the upper end of working hours (between 25 and 40 or more hours).

Other literature focused on child labour, productivity and the different ways these experiences interacted with school attendance and school performance; a Tanzanian cohort study examined child labour and future productivity saw a reduction in schooling and graduation rates, while research conducted in Lebanon found only "exclusive involvement in economic activity" negatively affected school attendance. In Brazil, children who reported working had higher school performance and less grade repetition. In Sri Lanka, as reported working hours increased, reports of regular attendance decreased although test scores reveal no differences between working and non-working students, while in Turkey the opposite situation was reported [2]. These differences in findings may be due differences in definitions of what constitutes long working hours, or excessive labour. Using a standard definition would improve comparability across studies and sites. This study supports concern being focused on work longer than 28 or more hours per week or paid work outside the home at lower numbers of hours per week, especially for younger children.

While there are distinct and specific negative associations between some kinds of work, school attendance and performance, there also appears to be an overall difference in school attendance, performance, and dropout rates between children who work long hours exclusively in market activity as compared to children who work long hours on household and domestic chores. Indeed, noted in the literature is a reasonable amount of non-market activity work (i.e., chores, light domestic work) is positively associated with better school performance, attendance, and perceptions of self worth [14,15,24,25].

While this manuscript with its focus on OAC adds to the growing body of literature on child labour, there are several important limitations to the study. First, data are reported by the caregivers and the authenticity of their responses cannot be validated. As reported in the literature, it is reasonable to assume that proclamations against child labour have created an atmosphere of anxiety around admitting to engaging in child labour [26-28]. While our interviewers succeeded in developing rapport with caregivers, we cannot rule out the possibility that their responses under-represent the extent of child work and labour practices. While the amount of child labour reported is within reason considering the reported rates for non-OAC in LMICs, it is plausible that the OAC population is at even greater risk of child labour. *The results from this study are not generalizable to non-OAC or OAC in "northern" or high income countries*.

Additionally, the literature would benefit from specific delineation of the activities that constitute chores, which this study did not do. In order to get at the core of how children are labouring and/or spend time in 'chore/household' activities, we need to collect more detailed information on daily activities. Importantly, because there is no information on the type of work being done, the analyses and child labour definition could not include hazardous work. Finally, the reference period for child work was the week prior to the interview, it is possible that children may engage in significantly different work or labour patterns during other times of the year, especially during the summer.

## Conclusions

While some kinds of child work or labour (household chores, part-time light work) can instil confidence, increase self-efficacy and contribute to family income, child labour is associated with low school attendance, and likely fewer occupational opportunities over time. This study found that the UNICEF definition of work for this age range is most associated with decreased schooling. While this relationship held when only hours of work were considered, it was stronger when work for pay was included. Lower numbers of hours worked were not significantly associated with decreased schooling.

High rates of child labour Among OAC suggest that the current policy focus on OAC due to the HIV global epidemic be expanded to protect OAC at risk of child labour. Identifying correlates of child labour that can help policy makers and local service providers identify families and children at risk, especially girls in households with little wealth and no income, in rural areas. This study supports the need to target support to families and households with OAC and not just the child.

## Competing interests

The authors declare that they have no competing interests.

## Authors' contributions

RW, KW, LM, JO and BP conceived of the analysis and interpreted the data. RW and KW conducted the literature review. LM and JO conducted the data analysis and editing. NT contributed to the overall drafting of the manuscript. MB provided technical and editing assistance. RW and KW conceived of the study. RW, KW, JO, NT and KO participated in the design and coordination of the study as well as acquisition of the data. RW drafted the initial manuscript and contributed to interpretation of data. LM contributed to variable design, performed the data analysis contributed to the drafting of the manuscript. JO contributed to variable design, performed the data analysis and edited the manuscript. KW contributed significant writing to the manuscript. BP contributed to variable design and conception of data analysis. All authors read and approved the final manuscript.

## Pre-publication history

The pre-publication history for this paper can be accessed here:

http://www.biomedcentral.com/1472-698X/11/1/prepub
